# High serum cholesterol predicts rheumatoid arthritis in women, but not in men: a prospective study

**DOI:** 10.1186/s13075-015-0804-1

**Published:** 2015-10-12

**Authors:** Carl Turesson, Ulf Bergström, Mitra Pikwer, Jan-Åke Nilsson, Lennart TH Jacobsson

**Affiliations:** Rheumatology, Department of Clinical Sciences, Malmö, Lund University, Inga-Marie Nilssons gata 32, Malmö, 205 02 Sweden; Department of Rheumatology, Skåne University Hospital, Inga-Marie Nilssons gata 32, 205 02 Malmö, Sweden; Department of Rheumatology, Eskilstuna Hospital, Kungsvägen 34, Eskilstuna, 631 88 Sweden; Department of Rheumatology and Inflammation Research, The Sahlgrenska Academy, University of Gothenburg, Guldhedsgatan 10, Gothenburg, 413 46 Sweden

**Keywords:** Rheumatoid arthritis, Cholesterol, Triglycerides, Predictors

## Abstract

**Introduction:**

Environmental exposures, including smoking, hormone-related factors, and metabolic factors, have been implicated in the etiology of rheumatoid arthritis (RA). A previous study has indicated that blood lipid levels may influence the development of RA. The objective of this study was to investigate the impact of serum total cholesterol and triglycerides on the risk of RA in a prospective study.

**Methods:**

Among participants in a large population-based health survey (*n* = 33,346), individuals who subsequently developed RA were identified by linkage to four different registers and a structured review of the medical records. In a nested case-control study, with controls, matched for age, sex, and year of inclusion, from the health survey database, the relation between serum lipids (levels of total cholesterol and triglycerides) and future RA development was examined.

**Results:**

In total, 290 individuals (151 men and 139 women) whose RA was diagnosed a median of 12 years (range of 1–28) after inclusion in the health survey were compared with 1160 controls. Women with a diagnosis of RA during the follow-up had higher total cholesterol levels at baseline compared with controls: odds ratio (OR) 1.54 per standard deviation; 95 % confidence interval (CI) 1.22–1.94. This association remained statistically significant in multivariate models adjusted for smoking and a history of early menopause and in analyses stratified by rheumatoid factor status and time to RA diagnosis. Total cholesterol had no significant impact on the risk of RA in men (OR 1.03; 95 % CI 0.83–1.26). Triglycerides did not predict RA in men or women.

**Conclusions:**

A high total cholesterol was a risk factor for RA in women but not in men. This suggests that sex-specific exposures modify the impact of lipids on the risk of RA. Hormone-related metabolic pathways may contribute to RA development.

**Electronic supplementary material:**

The online version of this article (doi:10.1186/s13075-015-0804-1) contains supplementary material, which is available to authorized users.

## Introduction

Rheumatoid arthritis (RA) is a chronic inflammatory disease of multifactorial etiology. Genetics [1] as well as environmental factors [2] have been implicated. Smoking is an established predictor of RA [3], and smoking and low socioeconomic status, as reflected by current occupation [4] or level of formal education [5], have been shown to have independent effects on disease development. Overall, women have a two- to three-fold higher incidence of RA than men [6]. We have previously demonstrated that women with early menopause (at age 45 or earlier) had an increased risk of RA compared with those with normal/late menopause in models adjusted for smoking, level of education, and length of breast-feeding, suggesting that hormone-related factors play a part in the pathogenesis [7].

Metabolic factors may also be important in this context. Recently, it has been suggested that obesity may influence both RA onset and disease severity [8]. Interestingly, the effect of obesity may be different in men and women, and findings indicate a reduced risk of RA in obese men [9, 10] and a positive association in women, in particular with obesity at a young age [11].

In a previous study of blood donors who subsequently developed RA and matched controls, average serum levels of total cholesterol (TC) and triglycerides (TGs) were higher in pre-RA cases than in controls [12]. This contrasts with studies of patients with established RA, in which lipid levels tend to be lower than in the general population, in particular in those with active, uncontrolled inflammation [13]. Furthermore, treatment with potent biologic anti-rheumatic drugs for severe, refractory RA has been associated with an increase in lipid levels, although the atherogenic index—ratio of low-density lipoprotein (LDL) cholesterol to high-density lipoprotein (HDL) cholesterol—appears to be unchanged in most cases [14, 15]. These patterns are of particular interest because of the increased risk of cardiovascular disease (CVD) in patients with RA compared with the general population [16], and data from some studies suggesting a “lipid paradox” in established RA, in which low TC and TG levels, probably related to active inflammation, are associated with CVD events [17].

The purpose of this study was to examine the effect of serum TC and TG levels on the risk of RA in a prospective study of men and women who were included in a large population-based health survey. To the best of our knowledge, this is the first prospective study to investigate this issue in women and men separately.

## Methods

### Source population

This nested case-control study used information from the Malmö Preventive Medicine Program (MPMP), a population-based health survey performed in Malmö, Sweden (current population: 300,000; during the screening period: approximately 235,000) in 1974–1992. The MPMP included a total of 33,346 individuals: 22,444 males born between 1921 and 1949 and 10,902 females born between 1925 and 1938. Details on the recruitment are described elsewhere [18]. The overall response rate was 71 %. During the first half of the period (1974–82), mostly men were invited, and mostly females during the second half (1982–92). The mean ages at screening were 49 years in women and 44 years in men. The vast majority of participants were Caucasians of Scandinavian origin.

### Exposure information

Blood samples were obtained in the morning, between 8 and 10 a.m., after an overnight fast. Serum TC and serum TG levels were immediately assessed by using fresh samples by enzymatic methods in routine use at the local hospital laboratory. The erythrocyte sedimentation rate (ESR) was measured at screening in accordance with the standard Westergren method. Height and weight were measured in light indoor clothing. Height was measured to the nearest centimeter, and weight was recorded at intervals of 0.1 kg. Body mass index (BMI) was calculated as weight (in kilograms) divided by height (in square meters).

Every participant filled out a self-administered questionnaire on medical and personal history, including smoking. For women who participated in April 1983 or later, the questionnaire included a series of questions on age at menopause. Similar to previous studies [7, 19], those with self-reported cessation of menstruations at age 45 or earlier were classified as having had early menopause and were compared with those with normal/late menopause (after 45 years of age). Women who were younger than 45 years at inclusion were excluded from analyses that included this parameter.

Data on self-reported overall health and self-reported cancer, diabetes, and cardiovascular disease (the latter classified as self-report of either hospitalization for stroke, physician diagnosis of angina pectoris or current use of heart medication) at baseline were extracted from the self-administered questionnaire. Data on socioeconomic status were derived from self-reported job titles in the Swedish national censuses, as previously described [4]. Briefly, occupations were coded and converted into standardized social class categories, and subjects were classified as “blue-collar workers” (manual workers, both skilled and unskilled), “white collar workers” (non-manual employees and self-employed professionals), and “others” [20].

### Cases and controls

As previously described [4], individuals who developed RA after inclusion in the MPMP and up to 31 December 2004 were identified by linking the MPMP databases to a community-based RA register [21, 22], the local outpatient clinic administrative register for Malmö University Hospital, the National Hospital Discharge Register [23], and the National Cause of Death Register [24]. In a structured review of all medical records, possible cases were validated and classified according to the 1987 American College of Rheumatology criteria for RA [25]. Only cases with a first documented diagnosis at least 1 year after inclusion in the MPMP were included. The index date was defined as the date of first diagnosis of RA in the index case. Four controls for each validated case, who were matched for sex, year of birth, and year of screening and who were alive, living in Sweden, and free of RA at the index date, were selected from the MPMP cohort. Vital status and information on emigration were retrieved from the national census, and controls who were not alive or living in Sweden through the index date were excluded.

Data on rheumatoid factor (RF) tests were retrieved from the two clinical immunology laboratories in the area. Patients with at least one positive test for RF were classified as RF-positive, whereas those tested who had only negative RF tests were classified as RF-negative.

### Statistics

The impact of baseline TC and TGs on the risk of RA was examined in bivariate conditional logistic regression analysis. Each case and the corresponding controls were given a group number, which was entered into the logistic regression models as a categorical variable. Odds ratios (ORs) per standard deviation (SD) of TC were calculated in men and women separately. To assess potential effect modification, the slopes for the estimates in men and women were compared, assuming a normal distribution. The impact of TC and TGs on the risk of RA was also assessed by entering the quartile of each of these potential predictors as a categorical variable in conditional logistic regression models in men and women separately. The second quartile, rather than the first, was used as the reference in these analyses, as low lipid values may reflect underlying inflammatory or catabolic conditions [26, 27]. Since TGs did not have a normal distribution, this was analysed only as a categorical variable and not as a continuous variable.

The impact of potential confounders on the risk of RA was examined in a similar manner. Associations between other exposures and serum levels of TC and TGs were assessed in linear regression models, adjusted for age, in men and women separately. Since the residuals in models including TGs did not have a normal distribution, the common logarithm of TGs was used as the dependent variable in the linear regression analyses.

Conditional logistic regression analyses were performed first bivariately and then adjusted for the other risk factors in multivariate analysis. Since data on menopause history were missing in 40 % of the female participants included in this study (Table 1), missing menopause data were included as a separate category in this analysis.

**Table 1:** Incident cases of rheumatoid arthritis and controls: characteristics and lipid levels, stratified by sex

	All		Women		Men	
	Cases	Controls	Cases	Controls	Cases	Controls
Number	290	1160	139	556	151	604
Age at inclusion	47.1 (7.1)	47.1 (7.1)	49.3 (7.4)	49.3 (7.4)	45.5 (6.2)	45.5 (6.2)
Years; mean (SD)						
Time from inclusion to RA diagnosis	12 (8–18)	NA	11 (7–16)	NA	13 (9–19)	NA
Years; median (IQR)						
Age at diagnosis	59.9 (8.8; 30–81)	NA	60.8 (8.4; 30–76)	NA	59.1 (9.0; 40–81)	NA
Years; mean (SD; range)						
RF-positive at diagnosis or later	179/250 (71.6 %)	NA	86/128 (67.2 %)	NA	93/122 (76.2 %)	NA
Current smokers at inclusion	155 (53.8 %)	486 (42.9 %)	64 (46.0 %)	197 (35.4 %)	91 (60.3 %)	289 (47.8 %)
Ever smokers at inclusion	181/260 (69.6 %)	625/1037 (60.3 %)	25/139 (61.2 %)	295/553 (53.3 %)	96/121 (79.3 %)	330/484 (68.2 %)
Blue-collar worker	146 (50.3 %)	496 (42.8 %)	67 (48.2 %)	230 (41.4 %)	79 (52.3 %)	266 (44.0 %)
White-collar worker	107 (36.9 %)	511 (44.1 %)	55 (39.6 %)	253 (45.5 %)	52 (34.4 %)	258 (42.7 %)
Early menopause (at age <46 years)*	NA	NA	24 %	17 %	NA	NA
Total cholesterol in mmol/l, mean (SD)	5.84 (1.11)	5.67 (1.04)	6.04 (1.16)	5.71 (1.10)	5.66 (1.02)	5.64 (0.98)
Triglycerides in mmol/l,	1.13 (0.78–1.57)	1.12 (0.83–1.55)	0.98 (0.76–1.48)	0.95 (0.72–1.31)	1.25 (0.90–1.70)	1.30 (0.98–1.77)
Median (IQR)						
BMI in kg/m^2^,	24.3 (3.5)	24.7 (3.7)	24.3 (4.1)	24.3 (4.3)	24.3 (2.7)	25.0 (3.3)
mean (SD)						
ESR in mm, median (IQR)	6 (4–11)	6 (4–10)	9 (5–14)	8 (5–12)	5 (3–8)	4 (3–7)
Full self-reported health	208/290 (72 %)	795/1155 (69 %)	99/139 (71 %)	364/551 (66 %)	109/151(72 %)	431/604 (71 %)
Self-reported cancer	8/260 (3 %)	28/1037 (3 %)	6/139 (4 %)	27/553 (5 %)	2/121 (2 %)	1/484 (0.2 %)
Self-reported diabetes	12/259 (5 %)	35/1036 (3 %)	9/138 (6 %)	25/562 (4 %)	3/121 (2 %)	10/484 (2 %)
Self-reported cardiovascular disease	8/260 (3 %)	27/1037 (3 %)	6/139 (4 %)	20/553 (4 %)	2/121 (2 %)	7/484 (1 %)

*SD* standard deviation, *RA* rheumatoid arthritis, *IQR* interquartile range, *NA* not applicable

*Data available from 84 cases and 334 controls

In addition, analyses were stratified by RF status at diagnosis or later (ever positive versus negative) and also by time from screening to RA diagnosis (above versus below the median) and by age at inclusion in women: younger than 46 years (i.e., usually pre-menopausal) versus at least 46 years. Statistical significance was set at a *P* value of less than 0.05 (two-sided test).

The study was approved by the regional research ethics committee for southern Sweden. All participants gave their informed consent to be included in the MPMP database. No informed consent was required specifically for the present study by the regional research committee.

## Results

### Incident cases and controls

RA was diagnosed in a total of 290 patients (151 men and 139 women) after inclusion in the MPMP. The median time from participation in the survey in which exposure data were collected to RA diagnosis was 12 years (range of 1–28 years), and patterns in men and women were similar (Table 1). The mean ages at inclusion were 49.3 years in women and 45.5 years in men. There were no substantial differences in ESR or self-reported health between cases and controls on the basis of data collected as part of the survey (Table 1). RA was diagnosed in six controls (2 men and 4 women) after the index date.

### Impact of lipid levels on the risk of RA-bivariate analyses

Women with a diagnosis of RA during the follow-up had higher TC levels at baseline compared with controls: OR 1.54 per SD; 95 % confidence interval (CI) 1.22–1.94. By contrast, there was no significant difference in TC levels between men who subsequently developed RA and controls (Table 2). The slope for the estimate for the impact of TC on RA development was significantly different between men and women (*P* < 0.001).

**Table 2:** Predictors of rheumatoid arthritis: bivariate conditional logistic regression analysis, stratified by sex

		Women	Men
		OR (95 % CI)	OR (95 % CI)
Current non-smoking		1.00 (ref.)	1.00 (ref.)
Current smoking		1.61 (1.04–2.49)	1.97 (1.29–3.02)
White-collar worker		1.00 (ref.)	1.00 (ref.)
Blue-collar worker		1.45 (0.97–2.27)	1.63 (1.05–2.53)
Normal/late menopause (at age ≥46 years)		1.00 (ref.)	NA
Early menopause (at age <46 years)*		1.67 (0.85–3.21)	NA
BMI, per SD		1.02 (0.84-1.28)	0.72 (0.58-0.89)
Total cholesterol, per SD		1.54 (1.22–1.94)	1.03 (0.83–1.26)
Total cholesterol, per quartile	Quartile 1**	1.19 (0.62–2.27)	0.84 (0.48–1.48)
	Quartile 2**	1.00 (ref.)	1.00 (ref.)
	Quartile 3**	1.74 (0.93–3.26)	0.92 (0.53–1.59)
	Quartile 4**	3.09 (1.67–5.72)	0.74 (0.42–1.32)
Triglycerides, per quartile	Quartile 1***	0.65 (0.36–1.17)	1.54 (0.80–2.69)
	Quartile 2***	1.00	1.00
	Quartile 3***	0.62 (0.34–1.15)	0.94 (0.53–1.67)
	Quartile 4**	1.23 (0.69–2.02)	0.93 (0.52–1.66)

*OR* odds ratio, *CI* confidence interval, *NA* not applicable, *SD* standard deviation, *BMI* body mass index

*Data available from 84 female cases and 334 controls

**Total cholesterol: quartile 1: women 2.9–4.9, men 3.1–5.0 mmol/l; quartile 2: women 4.9–5.6, men 5.0–5.6 mmol/l

Quartile 3: women 5.6–6.5, men 5.6–6.3 mmol/l; quartile 4: women 6.5–9.7, men: 6.3–9.4 mmol/l

***Triglycerides: quartile 1: women 0.30–0.73, men 0.18–0.96 mmol/l; quartile 2: women 0.73–0.95, men 0.96–1.29 mmol/l

Quartile 3: women 0.95–1.35, men 1.29–1.76 mmol/l; quartile 4: women 1.35–4.75, men: 1.76–5.32 mmol/l

Furthermore, women with TC in the highest quartile (6.5–9.7 mmol/l) had a significantly higher risk of developing RA compared with those in the second quartile (4.9–5.6 mmol/l) (OR 3.09; 95 % CI 1.67–5.72), and there was a similar trend for those in the third quartile (Table 2). The risk of RA among men in the highest quartile of TC levels was not increased compared with those in the second quartile (OR 0.74; 95 % CI 0.42–1.32). There was no significant impact of the quartile of serum TG levels on the risk of RA in women or in men, and no trend suggested a dose-dependent effect of TGs in prediction of RA (Table 2).

### Potential confounders

Smoking and blue-collar working status were predictive of RA in both sexes (Table 2). There was a trend toward an association between early menopause and subsequent development of RA, although it did not reach statistical significance in the present sample (Table 2). As we have reported previously [28], there was a negative association between BMI and future RA in men, whereas BMI had no significant impact on the risk of RA in women (Table 2).

Women with a history of early menopause had significantly higher TC levels compared with those with normal/late menopause (age-adjusted β 0.51; *P* < 0.001). Current smoking was associated with higher TC in women (age-adjusted β 0.20; *P* = 0.01) and there was a similar tendency in men (age-adjusted β 0.12; *P* = 0.09). Blue-collar workers, compared with white-collar workers, tended to have lower TC levels in men (age-adjusted β −0.09; *P* = 0.26). In contrast, there was no difference in TC depending on socioeconomic status in women (age-adjusted β 0.01 for blue-collar versus white-collar worker status; *P* = 0.89). There was a positive correlation between BMI and TC in women (age-adjusted β 0.02; *P* = 0.03) as well as in men (age-adjusted β 0.04; *P* < 0.001).

TG levels were higher in current smokers compared with non-smokers in women (*P* = 0.001) as well as in men (*P* = 0.03). Early menopause was associated with higher TG levels (*P* = 0.02). Blue-collar workers had lower TGs compared with white-collar workers in women (*P* = 0.004), whereas there was no such difference in men (*P* = 0.24).

### Lipids and RA-multivariate analyses

The positive association between higher TC levels and increasing risk of RA in women remained statistically significant in analyses adjusted for smoking (OR 1.47 per SD; 95 % CI 1.16–1.86) or early menopause (OR 1.45 per SD; 95 % CI 1.09–1.95) and also in a model adjusted for both covariates (OR 1.44 per SD; 1.13–1.83). In women, the risk of RA was significantly elevated among those with TC in the highest quartile, compared with the second quartile, in analysis adjusted for smoking as well as in the model that included both smoking and early menopause (Table 3). Results were similar in models further adjusted for socioeconomic status (Table 3). The impact of TC on the risk of RA in women remained significant in a model adjusted for BMI in addition to smoking, early menopause, and socioeconomic status (OR 1.42; 95 % CI 1.08–1.87).

**Table 3:** Impact of total cholesterol on the risk of rheumatoid arthritis in women: multivariate analyses

	Adjusted for smoking	Adjusted for smoking and early menopause	Adjusted for smoking, early menopause and socioeconomic status
	OR (95 % CI)	OR (95 % CI)	OR (95 % CI)
Total cholesterol, per SD	1.47 (1.16–1.86)	1.44 (1.13–1.83)	1.42 (1.08–1.86)
Total cholesterol–quartile 1*	1.11 (0.57–2.16)	1.12 (0.58–2.18)	1.44 (0.69–3.04)
Total cholesterol–quartile 2*	1.00 (ref.)	1.00 (ref.)	1.00 (ref.)
Total cholesterol–quartile 3*	1.71 (0.90–3.24)	1.70 (0.89–3.23)	2.34 (1.13–4.81)
Total cholesterol–quartile 4*	2.72 (1.44–5.14)	2.62 (1.37–4.99)	2.79 (1.22–5.87)

*OR* odds ratio, *CI* confidence interval, *SD* standard deviation

*Quartile 1: 2.9–4.9 mmol/l; quartile 2: 4.9–5.6 mmol/l; quartile 3: 5.6–6.5 mmol/l; quartile 4: 6.5–9.7 mmol/l

In men, there was no association between TC levels, either as a continuous variable or per quartile, and RA development in models adjusted for smoking alone or smoking, socioeconomic status, and BMI (Table 4).

**Table 4:** Impact of cholesterol on the risk of rheumatoid arthritis in men: multivariate analyses

	Adjusted for smoking	Adjusted for smoking, socioeconomic status and BMI
	OR (95 % CI)	OR (95 % CI)
Total cholesterol, per SD	1.01 (0.81–1.24)	1.02 (0.80–1.30)
Total cholesterol–quartile 1*	0.92 (0.52–1.62)	0.82 (0.43–1.57)
Total cholesterol–quartile 2*	1.00 (ref.)	1.00 (ref.)
Total cholesterol–quartile 3*	0.95 (0.54–1.65)	0.99 (0.53–1.85)
Total cholesterol–quartile 4*	0.75 (0.42–1.44)	0.80 (0.41–1.57)

*OR* odds ratio, *CI* confidence interval, *BMI* body mass index, *SD* standard deviation

*Quartile 1: 3.1–5.0 mmol/l; quartile 2: 5.0–5.6 mmol/l; quartile 3: 5.6–6.3 mmol/l; quartile 4: 6.3–9.4 mmol

TG levels had no significant impact on the risk of RA in multivariate analyses of women or men (data not shown).

### Stratified analyses

In women, there was a positive association between higher serum TC and increasing risk of RF-positive RA (OR 1.45 per SD; 95 % CI 1.08–1.94) as well as RF-negative RA (OR 1.85 per SD; 95 % CI 1.22–2.80). Patterns were similar to the main results for the analyses by quartile of TC (Additional file 1: Table S1). There were no associations between TC and the risk of RA in men in analyses stratified by RF status (Additional file 1: Table S2).

The risk of RA diagnosis during the follow-up increased significantly with higher TC in analyses of female cases investigated 1–12 years before RA diagnosis as well as among those investigated 13–28 years before diagnosis and their corresponding controls (Additional file 1: Table S3). In men, patterns were similar in analyses stratified by time to RA diagnosis in the cases, and there were no significant associations between TC and the risk of RA (Additional file 1: Table S4).

In a limited number of individuals (27 women and 20 men), RA was diagnosed within 5 years from inclusion. The impact of TC on the risk of RA was not substantially different from that observed in the entire sample in women (OR 1.60 per SD; 95 % CI 0.97–2.64) as well as in men (OR 0.69 per SD; 95 % CI 0.38–1.26). Finally, there was a significant association between TC levels and future RA among women who were younger than 46 years at inclusion as well as among women who were 46 years or older (Additional file 1: Table S5).

## Discussion

In this nested case-control study, women with a high serum TC were at an increased risk of developing RA in the future. By contrast, TC did not have any significant impact on the risk of RA in men. Serum TG levels were not predictive of RA.

Since there was no major change in these patterns in analyses adjusted for smoking and other potential confounders, it is unlikely that they are explained by such exposures. Rates of self-reported key co-morbidities were low and similar in cases and controls. Still, residual confounding by other factors remains a possibility.

Data on treatment with statins and other lipid-lowering therapy were not available in the present study. In the 1970s and 1980s, when most of the study participants were screened, such treatment was not commonly prescribed for primary prevention of CVD in Sweden. For example, the overall utilization of lipid-lowering drugs in 1987 was estimated to be 0.6 defined daily doses per 1000 inhabitants per day in a Swedish population-based survey [29]. Furthermore, baseline rates of self-reported CVD and diabetes in the present sample were low, further supporting that statin treatment at the time of inclusion was limited. However, subsequent prescription of lipid-lowering agents in individuals with high TC at inclusion in the MPMP could influence the present results.

It has previously been suggested that treatment with statins may be associated with an increased risk of developing RA [30]. This was based on a study using the Netherlands Information Network of General Practice, in which patients with RA were demonstrated to be more likely to have been prescribed statin treatment before diagnosis compared with matched controls, although there was no consistent impact of treatment duration or number of defined daily doses [30]. Another study found that, among individuals prescribed statins, those on persistent therapy had a reduced risk of RA compared with those with a lower proportion of days covered by statin prescriptions [31]. Taken together, this suggests that the relation between statin exposure and RA development is likely to be complex.

The observation that high TC was significantly associated with increased risk of RA in women only may reflect underlying mechanisms that are sex-specific (e.g., effects related to female sex hormones). It is well established that TC levels increase during menopause [32] and that women with premature menopause have increased TC levels compared with age-matched controls [33]. As expected, we found that TC levels were higher in women with a history of early menopause, and there was a trend toward an increased risk of RA in this subset, similar to previous reports [7, 34, 35]. However, the association between higher TC and RA development remained significant in models adjusted for history of early menopause. Still, we cannot rule out that hormone-related factors leading to increased TC levels explain the present results. For example, a history of short-term breastfeeding (≤6 months) has been associated with higher lipid levels, reduced insulin resistance, and abdominal obesity at follow-up 16–20 years after the last pregnancy [36], and we [37] and others [38] have previously shown that women who breast-feed their children for an extended time have a reduced risk of RA. Unfortunately, data on previous breastfeeding were not available in the studied cohort. The fact that significant associations were seen between TC and subsequent RA development in younger women (younger than 46 years old at inclusion), and in women included more than 13 years before diagnosis, suggests that long-term effects of metabolic pathways influence susceptibility to RA in women.

Obesity, which may play a differential role in men and women with RA [9, 28], may be important in this context. However, the estimated effects of TC in men and women were similar to the main results in models adjusted for BMI. Differences in body fat distribution and differences in the relation between biomarkers of inflammation and lipid metabolism in women and men may also contribute to this pattern and should be further studied. Our results are to some extent in agreement with those of van Halm et al., who reported higher TC in serial samples from pre-RA cases recruited from a population of blood donors investigated between 0.7 and 14.5 years before diagnosis [12]. However, the present study extends these findings to a population-based health survey of individuals from a more heterogeneous background than most cohorts of blood donors and to women included long before RA diagnosis. In contrast to the study by van Halm et al. [12], we found no association between TG levels and RA development. Potential explanations include methodological differences, such as the time of sampling (fasting in the present study).

In a retrospective study of a population-based cohort of patients with RA, serum TC and TGs decreased significantly compared with matched reference subjects during the last 5 years before RA diagnosis [39]. Data on prescribed lipid-lowering agents did not appear to explain these changes, which may be due to early effects of inflammation in the pre-clinical phase of RA. These observations, together with the considerable lag time from inclusion to RA diagnosis and the low ESR levels in most participants in the present study, suggest that our observations of higher TC in women who later developed RA were not due to such inflammatory mechanisms. Further studies of biomarker patterns in pre-RA cases with high TC may give insights into the underlying mechanisms and eventually be the basis for disease preventive strategies.

Current guidelines suggest monitoring of TC [40] or the TC/HDL cholesterol ratio [41] in patients with RA and the use of these parameters in risk score models, similar to those recommended for the general population [42]. However, there has been a lack of consistent associations between lipid levels and CVD co-morbidity in patients with established RA in several studies [43–45] and a paradoxical reverse association with TC and LDL cholesterol in a population-based sample of patients whose RA was diagnosed between 1988 and 2007 [17]. The present findings, suggesting an extended period of hypercholesterolemia before disease onset in some cases, are of interest for our understanding of atherosclerosis in the setting of RA. Further studies should examine the relation between pre-disease TC levels and CVD events after diagnosis.

Limitations of this study include the lack of data on HDL and LDL cholesterol as well as on apolipoproteins. Such data would be of particular interest, as women tend to have higher HDL cholesterol levels than men, although there is a decrease in HDL cholesterol and an increase in TC and LDL cholesterol after menopause [46]. Sex-specific effects of cholesterol fractions, as well as of lipid-lowering treatment, should therefore be further studied.

Owing to the study design, longitudinal data on lipids were not available. Therefore, we cannot assess the impact of changes in TC and TGs over time on the risk of RA. In addition, since the analyses were based on samples obtained between 1974 and 1992, effects of recent secular trends in lipid levels and related exposures in the population would not be identified. Further studies should include data on serum lipid levels from more recent health surveys and cases with RA onset in the last few years.

Another limitation is the lack of data on anti-citrullinated antibody (ACPA) status after diagnosis, which is due to the fact that for the majority of patients the diagnosis was before routine ACPA analysis was available. However, we did have data on RF, and RF positivity and ACPA positivity are known to have a major overlap in patients with RA [47]. Previous studies have indicated that predictors differ for RF/ACPA-positive versus RF/ACPA-negative RA [48]. For example, smoking has consistently been found to be a significant predictor of seropositive RA but not seronegative RA [5, 48, 49]. The positive association between higher TC and subsequent RA development in women was at least as strong for RF-negative RA as for RF-positive RA, suggesting distinct underlying disease mechanisms from those related to smoking.

The strengths of this study include the prospective study design, with exposures measured before RA diagnosis in a standardized manner. The low number of controls whose RA was diagnosed after the index date suggests that the potential impact of surveillance bias in patients with high TC and related diseases is limited. Furthermore, the inclusion of a relatively large sample of men who subsequently developed RA is a unique asset of this study. The population-based approach, with a high participation rate in the health survey, indicates that our cases were representative of patients with RA in the area. On the other hand, the cases were mainly Caucasians of Scandinavian heritage, and the results may not apply to other ethnic groups or other geographic settings.

## Conclusions

In this prospective study, there was a positive association between higher TC and increased long-term risk of RA in women but not in men. The impact of TC on subsequent disease development in women was significant for seropositive as well as seronegative RA. The estimates were similar in models adjusted for relevant confounders (i.e., smoking and early menopause). These findings suggest hormone-related metabolic pathways in the early pathogenesis of RA and may have implications for disease prevention and CVD risk management.

## Additional file

Additional file 1:
**Stratified analyses.** Impact of total cholesterol on the risk of RA, by sex and further stratified by RF status, time to RA diagnosis and age at inclusion in the cases. (DOCX 19 kb)

## Abbreviations

ACPA: Anti-citrullinated peptide antibody
BMI: Body mass index
CI: Confidence interval
CVD: Cardiovascular disease
ESR: Erythrocyte sedimentation rate
HDL: High-density lipoprotein
LDL: Low-density lipoprotein
MPMP: Malmö Preventive Medicine Project
OR: Odds ratio
RA: Rheumatoid arthritis
RF: Rheumatoid factor
SD: Standard deviation
TC: Total cholesterol
TG: Triglyceride
