# Supplementary material for: High serum cholesterol predicts rheumatoid arthritis in women, but not in men: a prospective study
Source: Arthritis Res Ther. 2015 Oct 12;17:284. doi: 10.1186/s13075-015-0804-1 (PMC4603637; doi:10.1186/s13075-015-0804-1)
Supplement: Additional file 1: — Stratified analyses. Impact of total cholesterol on the risk of RA, by sex and further stratified by RF status, time to RA diagnosis and age at inclusion in the cases. (DOCX 19 kb) [file 13075_2015_804_MOESM1_ESM.docx]

**Additional file 1**

**Stratified analysis**

**Table 1.** The relation between total cholesterol and the risk of RA in women, by RF status for the case.

Bivariate analysis

|  | RF positive RA  OR (95 % CI) | RF negative RA  OR (95 % CI) |
| --- | --- | --- |
| Total cholesterol,  per SD | 1.45 (1.08-1.94) | 1.85 (1.22-2.80) |
| Total cholesterol – quartile 1* | 2.40 (1.06-5.45) | 0.24 (0.06-0.95) |
| Total cholesterol – quartile 2* | 1.00 (ref) | 1.00 (ref) |
| Total cholesterol – quartile 3* | 2.02 (0.86-4.74) | 1.00 (0.36-2.81) |
| Total cholesterol – quartile 4* | 5.03 (2.18-11.6) | 1.72 (0.63-4.67) |

RF=rheumatoid factor OR=Odds ratio, CI=Confidence interval, SD=standard deviation

* Quartile 1: 2.9-4.9 mmol/l; Quartile 2: 4.9-5.6 mmol/l;

Quartile 3: 5.6-6.5 mmol/l; Quartile 4: 6.5-9.7 mmol/l

**Table 2.** The relation between total cholesterol and the risk of RA in men, by RF status for the case.

Bivariate analysis

|  | RF positive RA  OR (95 % CI) | RF negative RA  OR (95 % CI) |
| --- | --- | --- |
| Total cholesterol,  per SD | 1.04 (0.80-1.35) | 1.39 (0.82-2.38) |
| Total cholesterol – quartile 1* | 0.68 (0.34-1.34) | 0.80 (0.16-4.03) |
| Total cholesterol – quartile 2* | 1.00 (ref) | 1.00 (ref) |
| Total cholesterol – quartile 3* | 0.70 (0.35-1.42) | 1.38 (0.41-4.67) |
| Total cholesterol – quartile 4* | 0.65 (0.31-1.37) | 1.08 (0.31-3.81) |

RF=rheumatoid factor OR=Odds ratio, CI=Confidence interval, SD=standard deviation

* Quartile 1: 3.1-5.0 mmol/l; Quartile 2: 5.0-5.6 mmol/l;

Quartile 3: 5.6-6.3 mmol/l; Quartile 4: 6.3-9.4 mmol

**Table 3.** The relation between total cholesterol and the risk of RA in women, by time to RA diagnosis for the case.

Bivariate analysis

|  | 1-12 years  OR (95 % CI) | 13-28 years  OR (95 % CI) |
| --- | --- | --- |
| Total cholesterol,  per SD | 1.36 (1.02-1.80) | 1.94 (1.30-2.87) |
| Total cholesterol – quartile 1* | 1.06 (0.46-2.46) | 1.39 (0.49-3.94) |
| Total cholesterol – quartile 2* | 1.00 (ref) | 1.00 (ref) |
| Total cholesterol – quartile 3* | 1.13 (0.51-2.50) | 3.78 (1.30-11.0) |
| Total cholesterol – quartile 4* | 1.94 (0.89-4.23) | 6.98 (2.38-20.4) |

OR=Odds ratio, CI=Confidence interval, SD=standard deviation

* Quartile 1: 2.9-4.9 mmol/l; Quartile 2: 4.9-5.6 mmol/l;

Quartile 3: 5.6-6.5 mmol/l; Quartile 4: 6.5-9.7 mmol/l

**Table 4.** The relation between total cholesterol and the risk of RA in men, by time to RA diagnosis for the case.

Bivariate analysis

|  | 1-12 years  OR (95 % CI) | 13-28 years  (95 % CI) |
| --- | --- | --- |
| Total cholesterol,  per SD | 1.12 (0.83-1.51) | 0.94 (0.70-1.26) |
| Total cholesterol – quartile 1* | 0.85 (0.38-1.89) | 0.84 (0.39-1.84) |
| Total cholesterol – quartile 2* | 1.00 (ref) | 1.00 (ref) |
| Total cholesterol – quartile 3* | 0.95 (0.42-2.13) | 0.89 (0.42-1.88) |
| Total cholesterol – quartile 4* | 0.82 (0.35-1.93) | 0.68 (0.31-1.49) |

OR=Odds ratio, CI=Confidence interval, SD=standard deviation

* Quartile 1: 3.1-5.0 mmol/l; Quartile 2: 5.0-5.6 mmol/l;

Quartile 3: 5.6-6.3 mmol/l; Quartile 4: 6.3-9.4 mmol

**Table 5.** Impact of total cholesterol on the risk of RA in women, by age at inclusion for the case.

Bivariate analysis

|  | <46 years  OR (95 % CI) | ≥ 46 years  OR (95 % CI) |
| --- | --- | --- |
| Total cholesterol,  per SD | 1.75 (1.09-2.86) | 1.48 (1.15-1.93) |
| Total cholesterol – quartile 1* | 1.63 (0.73-3.60) | 0.59 (0.16-2.24) |
| Total cholesterol – quartile 2* | 1.00 (ref) | 1.00 (ref) |
| Total cholesterol – quartile 3* | 2.36 (0.98-5.71) | 1.17 (0.48-2.88) |
| Total cholesterol – quartile 4* | 4.50 (1.74-11.6) | 2.09 (0.92-4.72) |

OR=Odds ratio, CI=Confidence interval, SD=standard deviation

* Quartile 1: 2.9-4.9 mmol/l; Quartile 2: 4.9-5.6 mmol/l;

Quartile 3: 5.6-6.5 mmol/l; Quartile 4: 6.5-9.7 mmol/l
